# Current views and challenges on clinical cholera

**DOI:** 10.6026/97320630013405

**Published:** 2017-12-31

**Authors:** Charurut Somboonwit, Lynette J Menezes, Douglas A Holt, John T Sinnott, Paul Shapshak

**Affiliations:** 1 Department of Internal Medicine, University of South Florida, Morsani College of Medicine, Tampa, FL 33606, USA

**Keywords:** Cholera, Vibrio cholerae, diarrhea, vaccine, outbreak

## Abstract

Cholera, an acute diarrheal infection has become a major global threat. Vibrio cholerae the causative agent of cholera has been responsible for six previous pandemics since 1817 that spanned four continents and Australia with the seventh pandemic ongoing since 1961. Two serogroups of V. cholerae O1 and O139 have the ability to secrete the enterotoxin with potential to cause epidemics. The prior six pandemics were caused by the classical biotype of the O1 serogroup. However, the emergence of the El Tor biotype and subsequent variants of El Tor with classical traits are the main isolates in the seventh pandemic.

Cholera outbreaks have increased among vulnerable communities affected by war, earthquakes, conflicts and famines. Annually, 2.9 million cases of cholera occur globally in 69 endemic countries with 95,000 deaths. Early detection followed by prompt fluid and electrolyte replacement can reduce the case fatality ratio significantly. Improvements in water systems, sanitation and hygiene have effectively eliminated the transmission of cholera in high-income countries and reduced transmission in some developing nations. However, an estimated 1.8 billion are still at risk for cholera due to lack of potable water, inadequate sanitation and hygiene. Interventions focusing on hygiene in conjunction with proper disposal and treatment of sewage and provision of safe drinking water are likely to be effective in preventing the recurrence of cholera. Lastly, the use of current oral vaccines in endemic settings in combination with WASH interventions may be an effective approach to prevent and reduce the spread of cholera infection.

## Background

Cholera, caused by Vibrio cholerae, is an acute watery diarrheal illness that has become a major global health threat. There have been many V. cholerae epidemics. One such epidemic occurred in the Ganges Delta in the 1800s, spread beyond Asia as the first recorded pandemic in 1817 to include most of Africa, Europe, and Australia. Cholera spread to the Americas by the seventh pandemic that has been ongoing since 1961 [1, 2]. John Snow, during the third pandemic in 1853, observed that cholera was a waterborne infection [1, 3]. A year later, Fillipo Pacini identified V. cholerae, although he was not recognized for his discovery until 1966 [3]. The large-scale outbreaks that occurred over the course of seven documented pandemics were mostly caused by V. cholerae serogroup O1 classical biotype with the exception of one major outbreak in 1992 across Asia that saw the emergence of the O139 serogroup.

During the start of the current seventh pandemic in 1961, the El Tor biotype emerged and spread in most parts of Asia, swept west in the 1970s including Africa, the Middle East and the former USSR before arriving in Latin America in the early 1990s, most recently causing outbreaks in Hispaniola in 2010 [2]. Recent estimates indicate that the global burden of cholera is high with approximately 2.9 million cases and 95,000 deaths annually. Africa bears the largest burden representing 60% of cases and 68% of deaths. However, surveillance challenges and the widespread fear of political and economic implications have resulted in low reports of cases and deaths from affected countries [4].

## Epidemiology

Cholera - a preventable illness - is a reflection of the inherent social inequalities in low-and middle-income countries, and disproportionately affects the poorest and most vulnerable populations around the world. Humans are the only natural host for V. cholerae, which is transmitted via contaminated water or food. The bacterium has a relatively short incubation period of 2 hours to 5 days and generally requires a high inoculum dose (108 organisms) to cause disease in healthy individuals. The majority of infected are asymptomatic, however, infected individuals with symptoms of rice water stools might continue to shed the bacteria for up to two weeks or longer [5]. In 1961, the El Tor biotype, which was not previously implicated in epidemics, emerged from Sulawesi, Indonesia causing the seventh cholera pandemic. The El Tor biotype eventually became the predominant strain responsible for the annual cholera epidemics in India and Bangladesh. The 7th pandemic that began in 1961 became significant in Southeast Asia and in Africa. It also spread to Europe, North America, and Japan where the outbreaks were relatively limited due to improved sanitation. In 1991, the pandemic strain struck Peru then became widespread through most of the Western Hemisphere. Serogroup O139 emerged in India and Bangladesh in 1992 and for a while it appeared to be the dominant strain but has been quiescent since then, when the O1 serogroup El Tor biotype reemerged [6]. O139 has not been identified outside Asia [7]. Recent characterization of the El Tor biotype strains from the Haiti outbreak and in Bangladesh have revealed a variant that has incorporated Classical biotype traits producing increased amounts of cholera toxin as compared to the wild type El Tor biotype [8].

While safe drinking water and advanced sanitation systems have made Europe and North America cholera free for decades, cholera is currently endemic in 69 countries spanning the continents of Asia, Africa and the Americas with approximately 1.3 billion people at risk [4]. Cholera was conceptually classified as endemic or epidemic. These terms refer to two ends of the spectrum; however, there might be some overlap as epidemic cholera can occur in cholera endemic areas. Endemic cholera refers to the occurrence of fecal culture-positive cholera in a population for at least 3 of the past 5 years [9]. In endemic areas, children age 5 and under are the most susceptible due to low levels of pre-existing natural cholera immunity. In contrast, epidemic cholera is age independent and more severe as it is caused by the introduction of exogenous V. cholerae into a population that lacks pre-existing cholera immunity [10].

Since 2010, cholera continued to hit vulnerable communities affected by war, earthquakes, conflicts and famines [11]. For example, in 2015, such tragedies triggered reemergence of cholera in the Middle East, where some countries are already cholera endemic. Cross border movements especially between Iraq and its neighboring countries and locations with a high numbers of refugees are at risk for cholera epidemics [11]. Yemen, currently faces the world's largest cholera outbreak, with over 600,000 suspected cases and more than 2,000 deaths reported since April 2017. More than 800 people have died of cholera in Somalia since the beginning of 2017, and greater than 500 deaths have been reported in the Democratic Republic of Congo. Haiti, which was cholera free, has now reported an estimated 1 million cases with more than 10,000 deaths since the beginning of the 2010 outbreak post earthquake [12].

## Bacteriology and pathogenesis

V. cholerae is a comma shaped, motile, gram-negative bacillus with a single polar flagellum, belonging to the Vibrionaceae family. Its ecological niche is in salt or brackish water; and it is often associated with zooplankton and shellfish [13, 14]. More than 200 serogroups of V. cholerae have been identified. Until 1992, the V. cholerae strains that caused epidemic cholera because of their ability to secrete cholera toxin were categorized into two biotypes: classical and El Tor. Both biotypes contain two major serotypes, Inaba and Ogawa (and rarely Hikojima). They can be identified by agglutination in O group 1-specific antiserum directed against the lipopolysaccharide component of the cell wall and by demonstration of their enterotoxigenicity. The genes for cholera toxin are encoded by a filamentous bacteriophage, which differs slightly between classical and El Tor biotypes [2].

V. cholerae is a noninvasive intestinal pathogen. It is sensitive to acid, and most organisms are inactivated in the stomach. If the bacteria survive, they enter the small intestine where bile has role in preventing growth of the organisms by destabilizing the membranes and disrupting cellular homeostasis [15]. The surviving organisms will colonize the small bowel, where they secrete the potent cholera toxin, which causes excretory diarrhea. Cholera toxin in the small intestine induces intracellular cyclic AMP signaling via ADP-ribosylation of adenylate cyclase producing intestinal fluid secretion [16, 17]. Cholera toxin conjugated pillus facilitates colonization by quorum sensing [15]; Hemagglutinin/protease(HapA) and flagellum facilitate bacterial penetration through epithelial surface and mucous layers [18].

## Clinical manifestations

Secretory diarrhea, the hallmark of cholera, can lead to dehydration and electrolyte imbalances. The clinical features of cholera can range from mild to severe. The incubation period rages form 18 hours to 5 days. Diarrhea can be progressively watery with classic rice water, fishy odor diarrhea. Other symptoms such as vomiting, muscle cramps or ileus are common, however, in the presence of fever, work up for co-infections should be prompted. Severity of illness is usually related to the degree of dehydration [6]. In severe cases, dehydration resulting in death can occur within 6-12 hours after the onset of symptoms especially with absence of or delayed rehydration therapy. Severe dehydration might manifest as lethargy, a rapid radial pulse, loss of skin turgor, diminished urine output, low blood pressure, rapid breathing, sunken eyes and eventually hypovolemic shock [2, 3]. Complications are notable consequences of organ hypoperfusion (such as acute tubular necrosis and stroke) and electrolyte imbalances especially hypokalemia (abnormally low serum potassium causing fatigue, muscle weakness, ileus and arrhythmias) and hypernatremia (abnormally low serum sodium casing fatigue, muscle weakness and cramps, confusion), as well as hypoglycemia from insufficient liver gluconeogenesis. In severe cases, the patients can develop seizure from the above metabolic derangements [2].

## Diagnosis

Stool or rectal swab culture to isolate V. cholerae is the gold standard to confirm a diagnosis of cholera. The swabs are plated on selective alkaline peptone water enriched culture media, containing thiosulphate citrate bile salts, sucrose, or taurocholate- tellurite gelatin agar. Culture in non-selective media, needs to be confirmed later by slide agglutination with different monoclonal or polyclonal antibodies to detect V. cholerae O1 and O139 [19]. Dark field microscopy is about 50% sensitive by comparison to culture but can provide rapid detection of the bacteria due to its characteristic darting motility akin to "shooting stars"[20] which is inhibited by V. cholerae specific antibody [2, 21]. Low cost, rapid, point-of-care tests are important for the diagnosis of cholera in clinical and public health settings. However, to date, only a few tests are available, including the coagglutination test, the Sensitive Membrane Antigen Rapid Test, the Institut Pasteur and Crystal VC rapid dipsticks, and Medicos [2]. PCR assays are limited in utilization, and are available only in certain laboratory settings [2].

## Management

Prompt fluid and electrolyte replacement is the key to treat patients diagnosed with cholera. The initial assessment is to determine the level of dehydration and estimate the volume of body fluid that needs replacement. Cholera cot, a cot with a central hole for collection of diarrheal stool into a bucket, greatly assists measurement of stool output to reflect ongoing fluid loss. Otherwise, the ongoing fluid loss can be estimated as 10-20 mL/kg for each diarrheal stool or episode of vomiting [3, 22, 23].

Fluid replacement, either intravenous fluid or oral rehydration solution (ORS) can be used based on clinical presentation and degree of dehydration. Clinical assessment for degree of dehydration and fluid management are summarized in Table 1 [22]. Food should be avoided during the initial 4 hours of rehydration except breast-feeding to avoid vomiting. Antibiotics can help to reduce duration of diarrhea and risk of transmission. Antimicrobial options include macrolides, fluoroquinolones and tetracycline. A single dose azithromycin is typically preferred in the area where reduced susceptibilities to fluoroquinolones are common. However, azithromycin resistance emerged in the 1990's and became more common and concerning due to several resistance mechanisms including plasmid mediation and macrolide resistant proteins [24].

## Prevention

Improvements in sanitation, water systems and general hygiene have been key strategies in cholera control in industrial countries. Implementing such infrastructure in developing countries has neither been consistent nor always feasible due to the high costs. On October 4, 2017, the Global Task Force on Cholera Control (GTFCC) convened a high-level meeting of officials from choleraaffected countries, donors, and technical partners to affirm their commitment to ending cholera as a threat to public health by 2030 (The Global Roadmap to 2030). The strategy consists of three elements: "early detection and quick response to contain the outbreaks; a multi-sectorial approach to prevent cholera recurrence, and lastly, coordination of technical support and advocacy, resource mobilization and partnership at the global level". The ultimate goal of this strategy is to assist cholera affected countries to significantly decrease cholera deaths by 90% and eventually eliminate disease transmission in at least 20 countries by 2030 [12].

Effective cholera prevention and control measures are early detection and rapid control of outbreaks, universal use of potable water and basic sanitation, improving personal, food and water hygiene practices, quick access to treatment (intravenous fluid or Oral Rehydration Solution-ORS), and access to an efficacious oral cholera vaccine (OCV). Yet, the majority of cholera control activities have been focused on emergency responses to outbreaks, with limited attention to the underlying causes that can prevent future recurrence. For example, long-term WASH (improved water, sanitation, and hygiene) programs are too few and are not regularly prioritized in the areas most affected by cholera. Globally, WHO estimates indicate that there are 844 million people who lack access to a basic clean drinking water source, more than two billion drink water from fecally contaminated sources, and 2.3 billion are without basic sanitation facilities [7, 25].

The Global Roadmap to 2030 contains three strategic components. (1) Early detection, response and control of outbreaks by integrating early warning and surveillance system, re-positioning stocks of essential supplies (such as ORS, IV fluids, etc.), establish, implement and maintain of WASH system, set up dedicated health care facilities in the setting of outbreaks, improve health care facility infrastructure, OCV vaccination campaign, and community engagement to promote hygiene practices. (2) Prevention of disease occurrence by targeting cholera hotspots. This component not only identifies at risk areas but also implements certain control measures such as long-term WASH system, large scale use of OCV, and strong community and cross border engagement. (3) Effective technical support, resource mobilization and partnerships.

Despite clear goals, this initiative has several challenges including insufficient funding, lack of political will, unexpected events such as natural disasters and insufficient vaccine supplies. Besides setting cholera eradication as a priority, financial investment is needed to achieve the required WASH coverage in addition to ensure availability of OCV as well as maintenance of the programs [12].

Vaccine development and availability is the mainstay of cholera control in conjunction with improved sanitation. Intestinal mucosal immunity plays a key role of immune protection against cholera; and oral immunization as the most efficient approach to eliciting this immunity against cholera. Both live, genetically attenuated, and inactivated oral cholera vaccines have been developed. Although live attenuated vaccines have not been successful in protecting against cholera in endemic settings. WHO recommends the use of OCV preemptively in situations with high risk of cholera outbreak. There are two types of OCVs available: OCV that contains inactivated V. cholerae with cholera toxin B subunit (Dukoral®) or without cholera toxin B subunit (Shanchol™, Euvichol®, and mORCVAX™). These inactivated whole cell vaccines provide protection against cholera for at least three years [26], and they have been prequalified by WHO for purchase and use to prevent cholera in cholera-endemic populations. These vaccines are given as two or three-dose regimens. Vaxchora™, a live attenuated vaccine, CVD 103HgR, which was redeveloped after commercially discontinued; and it is the only FDA approved, single-dose oral vaccine for use by travelers aged 18-64 years [27]. In 2010, WHO recommended the use of oral cholera vaccines to control cholera outbreak in endemic settings. A working group was convened to create a global OCV stockpile for emergency response in 2013 [25]. The International Coordinating Group for vaccine provision received 12 requests containing 1.5 million doses of vaccines between 2014 and 2015. Requests were made for 1.5 million doses and 875,000 doses were delivered from the stockpile to affected countries such as South Sudan, Guinea, Haiti and The Democratic Republic of the Congo [22]. In large vaccination campaigns, using a single dose of OCV may prevent cases and deaths than a standard twodose campaign especially when vaccine supplies are limited [28, 29].

## Conclusions

Cholera has impacted human history. Our understanding of the bacteria, to protect the public has improved over the centuries. Despite the advent of improved sanitation, dehydration management, antimicrobial therapy and vaccine development, cholera remains a global threat. The current control strategies have a myriad of challenges and are ineffective in the areas with high burden of cholera. With political instability, increasingly displaced populations from wars, global warming, emerging variant of cholera strains; cholera control has again become a WHO priority. Ending Cholera-A Global Roadmap to 2030 was implemented. Equitable access and distribution of the OCV stockpiles should be available to population at risk and should follow the stockpile management mechanisms [30].

However, distribution of vaccine has to be done in conjunction with other measures to control and prevent the outbreaks. Identifying cholera hot spots and target vaccination will be cost effective in controlling outbreaks in endemic areas. Additionally, vaccination can be used to prevent cholera outbreaks in the setting of humanitarian crises. The development of improved new generations of OCVs, cost-effectiveness of OCV campaigns, new vaccine delivery strategies and social mobilization will enhance cholera control efforts. However, there are several important questions to be answered especially in resource distribution and sustainability of control measures in the affected areas. Collaborations among scientific, medical, public health, environmental, financial, and political institutions are crucial. Inequality remains largely at the core of the cholera disease burden, and it will continue to be a disease of major public health concern.

## Figures and Tables

**Table 1:** Assessment of diarrheal patients with dehydration and rehydration therapy. (Adapted from WHO. The treatment of diarrhea: a manual for physicians and other senior health workers (4th revision). Geneva: World Health Organization, 2005) [22].

Clinical Presentations:	No Dehydration	Some Dehydration	Severe Dehydration
General appearance	Well, alert	Restless, irritable	Lethargic or unconscious
Eyes	Normal	Sunken	Sunken
Thirst	Not thirsty	Thirsty, drinks eagerly	Drinks poorly or unable to drink
Skin tugor	Normal, goes back quickly after pinching	Poor, goes back slowly after pinching	Very poor, goes back very slowly after pinching
Fluid deficit (% body weight)	<5%	5-10%	>10%
Rehydration Therapy	Home therapy with ORS or salted drink, replace ongoing loss	Fluid (intravenous +/- ORS initially then ORS) 50-100 ml/kg and replace ongoing loss	Fluid (intravenous initially, then ORS) 100 ml/kg and replace ongoing loss
